# Is the Hippocampus a Potential Target for the Modulation of Mind Wandering in Major Depression?

**DOI:** 10.3389/fpsyt.2018.00363

**Published:** 2018-08-10

**Authors:** Juergen Fell

**Affiliations:** Department of Epileptology, University of Bonn, Bonn, Germany

**Keywords:** mind wandering, brain stimulation, hippocampus, major depressive disorder, mood disorders

Mind wandering refers to a state where attention is withdrawn from the task or situation currently at hand and drifts to internal thoughts, feelings or perceptions. This ubiquitous state has been reported to occupy up to half of the waking time of individuals (1). Until quite recently research has largely neglected this phenomenon, underlining why mind wandering has been referred to as the dark matter of cognitive neuroscience [e.g., (2)]. Mind wandering can be experimentally accessed, for instance, by thought probes and behavioral markers (e.g., errors, reaction times) during sustained attention tasks. It can have positive and negative effects (3). Amongst the positive effects are enhanced creativity and better future planning. The negative effects range from errors in ongoing perceptual and cognitive tasks to increased stress levels and a decline in mood. Since reduced mood conversely causes more frequent episodes of mind wandering (4) this mutual interdependence constitutes a vicious circle.

Rumination is a subtype of mind wandering and is a characteristic of patients suffering from major depressive disorder (MDD). Rumination is defined as repetitive, often obsessive thoughts with similar content. In depressive subjects these thoughts are often concerned with negative issues originating from past experiences (5). Rumination has been linked to the onset (6) and maintenance of depression (7), as well as to its severity. From 11 studies reviewed by Morrison and O'Conner (8) 10 reported that increased rumination is associated with increased suicidality. It has been proposed that mind wandering, which is negatively valenced, thematically homogeneous, and associatively narrowed is maladaptive and gives way to rumination leading to depressive symptoms (9). The relationship between mind wandering and depression has recently been experimentally quantified. Hoffmann and colleagues (10) used a non-demanding task requiring continuous attention, which from time to time was interrupted by so-called “experience sampling” probes. These probes consist of questions inquiring as to whether the subject's attention was focused on the task or not, and the content of their thoughts. Patients suffering from MDD reported more mind wandering episodes than healthy controls and that their thoughts were less positive, more past-oriented and self-related.

Hence, methods reducing the propensity or modulating the content characteristics of mind wandering represent potential therapeutic approaches in MDD. One approach is trying to enhance mindfulness or, in other words, meta-awareness of inner experiences, for instance, in the context of mindfulness-based cognitive therapy (11). However, mindfulness training is demanding, requires disciplined practice, which is beyond the capabilities of some patients, and can produce adverse effects [e.g., (12, 13)]. Brain stimulation techniques may provide an alternative option. But is there any evidence that mind wandering can be modulated using brain stimulation? Mind wandering has been shown to be related to activity in default mode network (DMN) and executive control regions (14, 15). Recently, two studies applying transcranial electrical stimulation provided evidence for a modulation of mind wandering (16, 17). Both studies targeted the dorso-lateral prefrontal cortex (DLPFC), an executive control region, one via anodal [(16), DLPFC], the other via cathodal stimulation [(17), LPFC], which are thought to increase/decrease cortical activation respectively [e.g., (18)]. Indeed, under anodal stimulation of the DLPFC, the propensity to mind wander was reported to increase (16), whereas under cathodal stimulation, a decline in mind wandering was observed (17). However, it remained unclear in these studies, whether the propensity to mind wander or just meta-awareness of the mind wandering episodes was modulated, which is indeed a prerequisite for reporting mind wandering and may also be related to DLPFC activity (19). Moreover, the DLPFC may be involved in the later, rather than in the early stages of mind wandering, e.g., in the elaboration and evaluation of the thoughts (20, 21). The most effective approach to modulate mind wandering, therefore, would be to target the region responsible for initiating mind wandering.

The big question is: which brain region initiates mind wandering? For the first time, a recent fMRI study has shed light on this question (22). In this study participants with long-term experience in mindfulness meditation (> 3,000 h) were instructed to immediately signal to the investigator the arising of spontaneous thoughts. The methodological strength of this study lies in the fact that subjects highly experienced in the observation of thought processes were recruited, which are capable of instantly recognizing the onset of thoughts—a rather difficult or impossible task for untrained subjects. Ellamil and colleagues found that activity in default mode and executive control regions, as well as in regions associated with the monitoring of inner experiences, were related to the occurrence of the spontaneous thoughts. Most interestingly, the region which was activated first, even before subjects signaled the onset of a thought, was the hippocampus (i.e., a default mode region), which is mainly known for its role in memory processes. The authors speculated that the hippocampus is well-suited to initiate spontaneous thoughts due to its capacity for novel connections at the synaptic level and its dense links to other brain areas (21, 22). Moreover, hippocampal activity is crucial for remembering past events and envisioning the future, which are typical contents of mind wandering [e.g., (23)]. Indeed, mental time travel during mind wandering has been shown to be accompanied by increased interaction between the hippocampus and other DMN regions (24).

Evidence for an involvement of the hippocampus in mind wandering also comes from a recent study investigating subjects with bilateral hippocampal damage (25). Based on a small sample [6 patients vs. 12 controls) the authors have reported that these patients engaged in as much mind wandering (decoupled spontaneous thoughts) as control participants. However, average mind wandering was numerically smaller in the patients and the hypothesis of decreased mind wandering was rejected based on a rather low *p*-value (two-tailed *p* = 0.12). Importantly, mind wandering of the patients was less past, but more present oriented and less visual-episodic, but more verbally-semantic. Other studies have reported a prominent contribution of hippocampal activity patterns to rumination in MDD (26) and altered DMN-hippocampus connectivity after sad memory-recall in remitted-MDD (27). Furthermore, Golchert et al. (28) have described structural magnetic resonance imaging differences associated with individual variations in the intentionality of mind wandering (i.e., spontaneous vs. deliberate). They found that spontaneous mind wandering is related to increased cortical thickness in the retrosplenial cortex, which is a node region for information transfer between the hippocampus and prefrontal, parietal and occipital regions [e.g., (29)]. They interpreted these results as indicating “that the spontaneous onset of mind wandering occurs when activity in the hippocampus is integrated into an ongoing train of thought via the retrosplenial cortex.” In line with these arguments, a recent fMRI study has found that the subgenual anterior cingulate cortex (sgACC), a region involved in the control and modulation of mood, becomes decoupled from the hippocampus and the retrosplenial cortex after administration of ketamine (30). Ketamine, a dissociative anestetic, is known to have rapid-acting antidepressant properties (31). Wong and colleagues (30) interpreted their results as being related to the reduction of mind wandering and depressive rumination. In accordance with this interpretation, a metanalysis of neuroimaging studies has shown that functional connectivity between the subgenual prefrontal cortex (including the sgACC) and the DMN is increased in major depression and often predicts levels of rumination (32). Taken together, the studies described above, in my opinion, point into the direction that the hippocampus is a core or even initiating region for mind wandering and rumination.

Could the hippocampus be targeted using brain stimulation in order to modulate the content characteristics or reduce the propensity of mind wandering in MDD? With regard to electric stimulation, the hippocampus cannot be easily accessed by the transcranial technique. Thus, the impact of conventional electric stimulation on the hippocampus can only be directly investigated in patients, in which intracranial electrodes are implanted, for instance, for the presurgical evaluation of epileptic seizure onset zones (33). However, several non-invasive brain stimulation approaches have recently been developed which may allow targeting of the hippocampus. One such method is pulsed transcranial ultrasound stimulation, which has been shown to be able to modulate hippocampal activity in mice (34). Recently, this technique has been successfully applied to humans for the modulation of activity in primary somatosensory (35) and visual cortex (36). Another technique is transcranial electric stimulation with temporally interfering electric fields (37). This technique uses the superposition of high-frequency electric fields comprised of slightly different frequencies, resulting in a low-frequency amplitude modulation. These interfering electric field patterns produce a spatially focused form of deep brain stimulation. It has been demonstrated in mice that the hippocampus can be selectively stimulated with this technique (37). However, the safety and transfer of this method to humans still has to be proven. In the auditory domain a similar technique has been established: auditory beat stimulation. This stimulation either consists of the superposition of two nearby frequencies identically applied to both ears (monaural beats), or the application of each frequency separately to each ear (binaural beats). Research in presurgical epilepsy patients has shown that hippocampal activity can be modulated by this kind of auditory stimulation (38).

Of course, there may be negative side effects of hippocampal stimulation, which should be considered. One potential side effect could be, for instance, that brain stimulation reducing hippocampal activity may not only decrease mind wandering, but also affect memory functioning. Since memory deficits are common in MDD—as are deficits across most cognitive domains [e.g., (39)], an additive stimulation-related memory impairment may be hard to tolerate. Another aspect to consider is that hippocampal functioning may contribute to the recovery from depression by supporting cognitive flexibility (40). Hence, the time period of hippocampal stimulation with regard to a possible transition from maladaptive to adaptive mind wandering (9) may be an important issue.

Is there evidence for a correlated modulation of rumination and hippocampal activity by antidepressant medication? Although it has been reported that treatment with selective serotonin-reuptake inhibitors (SSRIs) and serotonin-noradrenalin-reuptake inhibitors (SSNRIs) reduces rumination in major depression [e.g., (41)] and dysthymia [e.g., (42)], it is yet unknown whether certain antidepressants specifically target rumination. With regard to emotional processing, a meta-analysis of 9 fMRI and PET studies revealed decreased activation of the hippocampus and other limbic regions following antidepressant drug treatment mostly using SSRIs and SSNRIs (43). However, emotional processing is not always accompanied by rumination [e.g., (44)]. Regarding resting state network dynamics a single-dose of the SSRI sertraline in healthy subjects reduced functional connectivity (fMRI) between the limbic system, including the hippocampus, and the executive control network (45). Furthermore, a graph theory analysis of resting state functional connectivity (fMRI) in MDD patients vs. controls showed decreased nodal efficiency of the left hippocampus and an increase of nodal efficiency after treatment with antidepressants associated with an improvement of depression scores (46). The relation of these network changes to rumination, however, is not obvious. Thus, direct evidence for a correlated modulation of rumination and hippocampal activity by antidepressant medication is yet lacking.

In summary, the reduction or modification of contents of mind wandering is a potential therapeutic approach for the treatment of major depression. Recent findings suggest that the hippocampus is a core structure involved in, or even initiating mind wandering. Several non-invasive brain stimulation techniques have been developed, which may allow for the modulation of hippocampal activity. Therefore, I propose to explore whether a reduction of the propensity or modification of the content characteristics of mind wandering via modulation of hippocampal activity can be achieved with these techniques. If so, this line of research may open a new avenue for the treatment of major depression.

## Author contributions

The author confirms being the sole contributor of this work and approved it for publication.

### Conflict of interest statement

The author declares that the research was conducted in the absence of any commercial or financial relationships that could be construed as a potential conflict of interest.
